# Remote Sensing of Vital Signs: A Wearable, Wireless “Band-Aid” Sensor With Personalized Analytics for Improved Ebola Patient Care and Worker Safety

**DOI:** 10.9745/GHSP-D-15-00189

**Published:** 2015-09-10

**Authors:** Steven R Steinhubl, Mark P Marriott, Stephan W Wegerich

**Affiliations:** ^a^​ Scripps Translational Science Institute, La Jolla, CA, USA; ^b^​ Rhythm Diagnostic Systems, Palo Alto, CA, USA; ^c^​ physIQ, Chicago, IL, USA

## Abstract

This wireless sensor technology, currently being field-tested in an Ebola Treatment Unit in Sierra Leone, monitors multiple vital signs continuously and remotely. When connected with enhanced analytics software, it can discern changes in patients’ status much more quickly and intelligently than conventional periodic monitoring, thus saving critical health care worker time and reducing exposure to pathogens.

Continuous, remote monitoring of multiple vital signs in patients with Ebola, coupled with personalized data analytics, can warn health care workers of critical changes in patients’ status much earlier than with conventional intermittent monitoring—which typically occurs just every 8 hours—and without risking the safety of health care workers. This sophisticated technology, in turn, could lead to earlier initiation of lifesaving interventions, better health outcomes, and reduced risk of spreading the virus.

A consortium of academic and industry partners, STAMP^2^ (which stands for Sensor Technology & Analytics to Monitor, Predict and Protect Ebola patients), has developed such a novel, wearable patient sensor—resembling a band-aid—that tracks and wirelessly transmits multiple vital signs to remote health care workers in non-red zone observation areas. The technology, which includes the MultiSense sensor coupled with state-of-the-art, real-time data analytical capabilities, called Personalized Physiology Analytics (PPA), sends patient-specific automated alerts of any important changes in the patient’s condition without requiring the health care worker to constantly monitor display screens.

The technology was developed in response to the Grand Ebola Challenge, sponsored by the United States Agency for International Development (USAID), to develop innovative solutions to improve the safety of health care workers and care of patients with, or at high risk for, Ebola virus infection.

## MULTISENSE: WIRELESS, WEARABLE SENSOR

**FIGURE 1:**
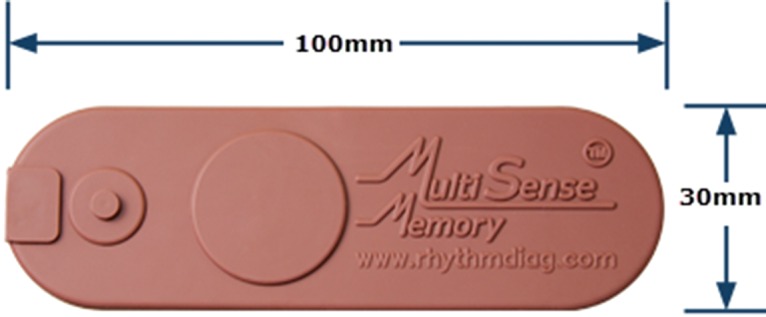
MultiSense Patient Sensor: Small, Wearable, and Wireless or via USB

**FIGURE 2:**
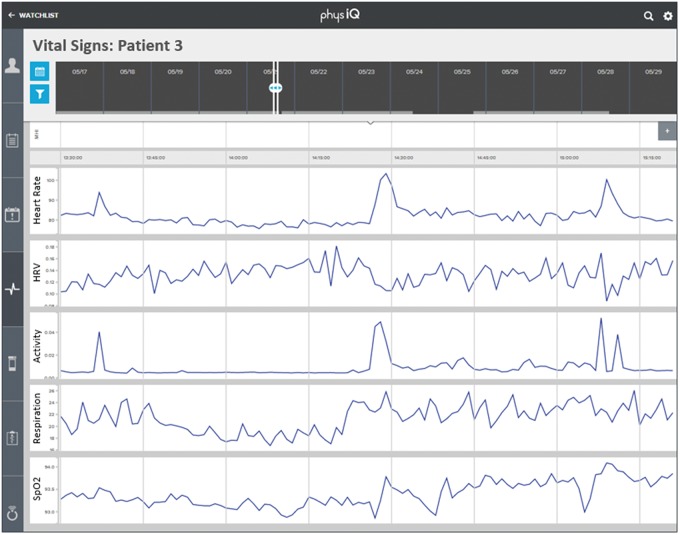
Illustrative Data Output for an Individual Patient From MultiSense Patient Sensor Top to bottom: heart rate, heart rate variability (HRV), activity level, respiration rate, and SpO2 (oxygen saturation).

There has been tremendous growth over the last several years in the field of mobile health (mHealth), and especially in wearable, wireless sensors.^1^ One example of a state-of-the-art wearable sensor is the MultiSense patch being developed by Rhythm Diagnostic Systems. MultiSense is a battery-powered, flexible strip, measuring 4 X 1.2 inches and weighing less than 15 grams (Figure 1). It is waterproof with a patented adhesive for reliable adherence to the chest in most situations. The patch has a proprietary low-noise electrocardiogram (ECG) design and is able to simultaneously track and record a number of physiological parameters beyond the single-lead ECG, including (Figure 2):

- Heart rate
- Pulse synchronized oxygen saturation
- Temperature
- Respiratory rate
- Depth of respiration
- Motion/position

The ability to reliably identify relative changes in blood pressure is also currently being optimized. The second-generation MultiSense patch is Bluetooth-enabled and can stream the data continuously, in real-time, to a smart phone or other device via Bluetooth LE. It has a low overall cost of manufacturing and is disposable. The patch is designed to be used on a single individual for 60+ days of monitoring, with adhesives changed as appropriate (typically every 4–10 days).

## PPA SOFTWARE: PERSONALIZED PHYSIOLOGY ANALYTICS

Coupled with the PPA data analytics technology, MultiSense provides a detailed snapshot of patients’ dynamic physiologic status based on their measured parameters and how they interact with each other. The analytics software, developed by physIQ, uses machine-learning algorithms that are purely data driven rather than based on any fixed set of parameters. Thus, the PPA software can be applied to a wide range of vital signs parameters including those measured by the MultiSense.

PPA is used to detect subtle changes in an individual’s physiological characteristics from learned baseline physiological behavior. Importantly, PPA detects changes in the interrelationships between parameters, which can be missed when examining parameters individually against population statistics. These detected deviations are generally within the normal range of variation seen in an individual’s vital signs, thus making it possible to detect change well in advance of symptoms or decompensation when an adverse health condition progresses, such as with Ebola infection.

**FIGURE 3:**
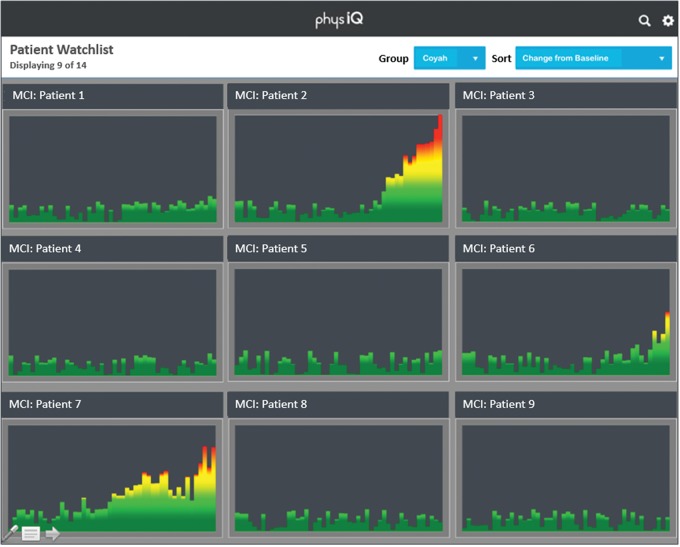
Illustrative Patient Watch List Displaying Multivariate Change Index (MCI)^a^ Trends for Multiple Patients ^a^ The MCI is a single derived measure of a patient’s status based on continuous tracking of a series of differences from the patient’s current vital signs compared in real-time with the patient’s baseline vital signs, representing the change in a patient’s physiology over time.

Using a component of PPA called Similarity Based Modeling, each patient’s current vital signs are compared with the patient’s baseline in real-time, to develop a series of residuals.^2^^,^^3^ These residuals are then fused into a single Multivariate Change Index (MCI)—a combined, personalized index that is much more sensitive than any one vital sign. The MCI represents the change in the patient’s physiology over time, identifying the early signs of a developing or worsening medical condition. Further, health care workers can view the MCIs of all monitored patients simultaneously, as shown in Figure 3, providing exception-based, efficient monitoring of a large population by limited clinical staff, especially important in low- and middle-income countries where health care workers are commonly a scarce resource.

## CURRENT STATUS AND NEXT STEPS

STAMP^2^ solution, after receiving approval from the Sierra Leone Ethics Committee, is currently undergoing field testing in Sierra Leone in collaboration with the International Medical Corps. Once validated in a population admitted to an Ebola Treatment Unit for suspected Ebola infection, a number of other uses are possible and can be evaluated. For example, the technology can be used to remotely monitor individuals exposed to Ebola, such as family members of patients, who are considered to be at especially high risk. In addition, similar technologies can be used to monitor health care workers directly, for example, to monitor early signs of heat stress while deployed in the field, as well as for more personalized, passive but continuous monitoring after returning home to detect important changes prior to the development of symptoms or to detect the earliest signs of temperature elevation not dependent upon population-based averages.^4^

## Notes

**Peer Reviewed**
